# Molecular evidence supports the functionality of a protein-trapped endogenous allele of Dally-like protein.

**DOI:** 10.17912/micropub.biology.001283

**Published:** 2025-01-25

**Authors:** Drew Delmore, Indrayani Waghmare

**Affiliations:** 1 Department of Biological Sciences, University of Massachusetts Lowell, Lowell, Massachusetts, United States

## Abstract

The *Drosophila* glypican Dally-like protein (Dlp) is an evolutionarily-conserved cell-surface protein that modulates extracellular distribution of several secreted ligands for cell signaling. Several fly lines expressing tagged *dlp* have been used to study the role of Dlp *in vivo* including the *PBac{602.P.SVS-1}dlp ^[CPTI000445]^* protein-trap line, which encodes StrepII-Venus-StrepII (SVS)-tagged Dlp from the endogenous locus. *dlp* is essential for embryonic development, and the *SVS-dlp* line is homozygous viable. Although this suggests that the SVS-tagged Dlp is functional, it is possible that that the *SVS-dlp* flies produce wild-type *dlp* isoform through alternative splicing, contributing to their survival. Here, we used a molecular analysis approach to show that the *SVS-dlp* flies do not produce wild-type isoform, confirming that the SVS-tagged Dlp is indeed functional.

**Figure 1. Molecular analysis of the SVS-dlp protein trap line:**
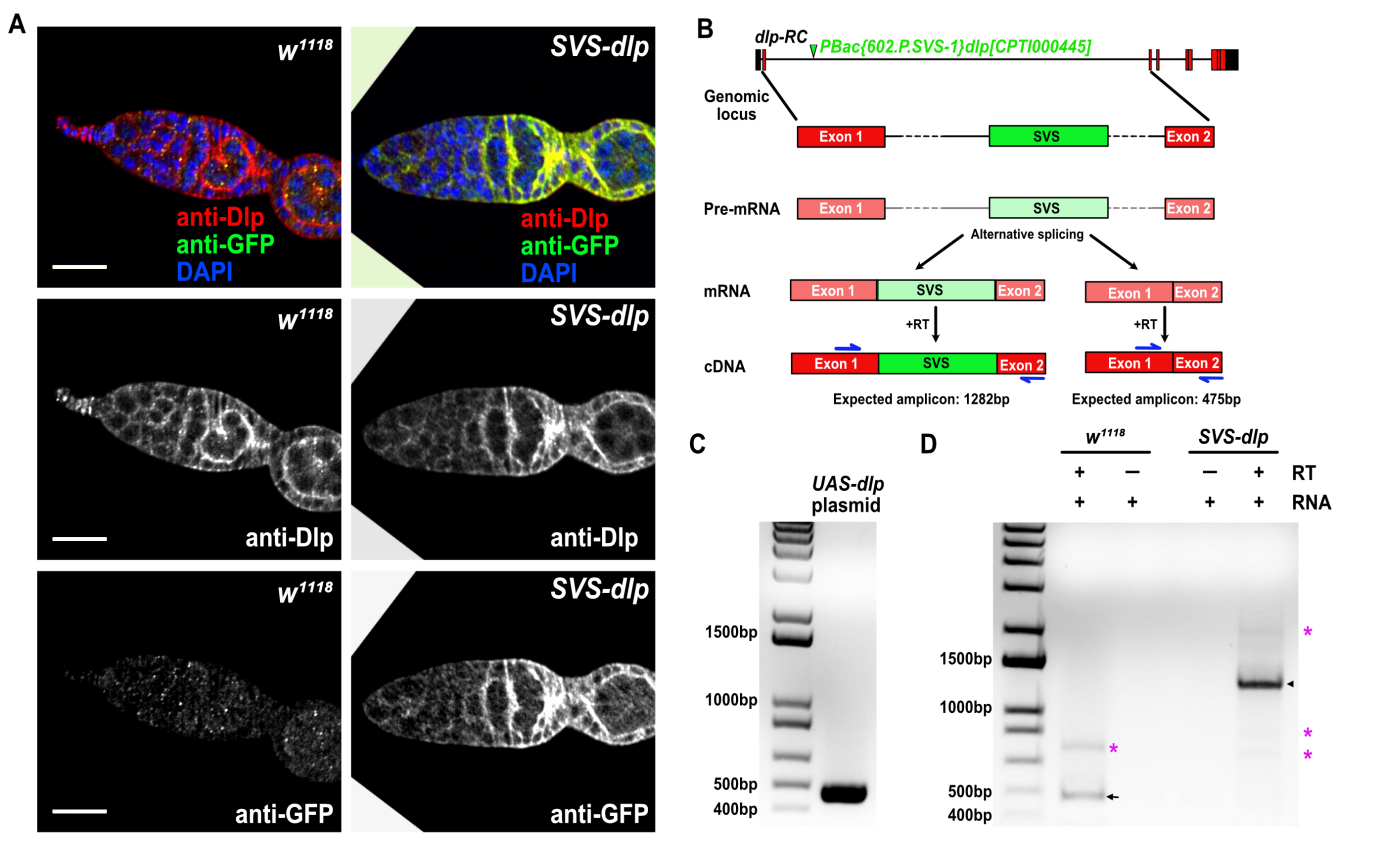
A) Germaria from age-matched *w ^1118^* and *SVS-dlp* flies were stained with anti-Dlp and anti-GFP antibodies. Anti-Dlp detects epitopes on the C-terminal region of Dlp, and anti-GFP detects epitopes on the Venus tag. Anti-Dlp staining is detected in tissues isolated from both *w ^1118^ and SVS-dlp* flies but anti-GFP staining is detected only in samples isolated from *SVS-dlp* flies. In *SVS-dlp* flies, anti-Dlp and anti-GFP stainings colocalize. A single comparable Z section is shown. B) Schematic representation of the location of the *PBac{602.P.SVS-1}dlp ^[CPTI000445]^* insertion in the endogenous *dlp* locus in the protein trap line and the experimental strategy to assess if the insertion produces functional SVS-tagged Dlp. *PBac{602.P.SVS-1}dlp ^[CPTI000445]^* is located in the first intron and contains an artificial exon encoding SVS. Two possible *dlp* mRNA isoforms may arise via alternative splicing of the pre-mRNA in the protein trap line, one containing the artificial SVS exon and one without. When reverse transcribed, the cDNA serves as a proxy for the presence of these isoforms due to substantial difference in length. We used primers (blue) against the sequences in exon and 1 and 2 to detect these two isoforms. The isoform containing the SVS artificial exon is expected to generate a 1282bp amplicon whereas the isoform without the SVS artificial exon is expected to generate a 475bp amplicon, which indicates presence of the wild-type isoform without the artificial exon. C, D) Results from DNA gel electrophoresis are shown. C) Standard PCR was performed using *UAS-dlp* plasmid as a template, and a single 475bp amplicon is observed. D) Total RNA was isolated from *w ^1118^* (control) and *SVS-dlp* adult males and females followed by incubation with reverse transcriptase (RT) to generate cDNA. RNA samples not incubated with reverse transcriptase are negative controls. A 475bp amplicon (black arrow) is detected in sample isolated from *w ^1118^* flies but not in sample isolated from *SVS-dlp* flies. A1282bp amplicon (black arrow) is detected in sample isolated from *SVS-dlp* flies. Several high molecular weight non-specific bands, marked by pink asterisk, are also observed both in control and *SVS-dlp* flies, but only in the presence of reverse transcriptase. These are non-specific bands that likely arise from amplification of regions of other cDNA templates generated in the sample by reverse transcriptase as these bands are absent when *UAS-dlp* plasmid is used as a template. These non-specific bands, however, do not change the interpretation of the results as the goal of the experiment was to determine whether or not the *SVS-dlp* flies produce wild-type isoform, which they do not. Images in C and D are qualitatively comparable. Scale bar in A: 20 microns.

## Description

Cells in multicellular organisms communicate with each other to ensure proper tissue development and homeostasis. The intercellular communication is typically orchestrated by several secreted ligands such as Wnts, Hedgehog, and Decapentaplegic that are produced by source cells to activate signaling in target cells, which may be located at short or long range (Lawrence and Struhl, 1996; Neumann and Cohen, 1997) . The extracellular distribution of the secreted ligands from the source cells partly depends on a cell-surface glypican called Dally-like protein (Dlp), which performs an evolutionarily-conserved function of interacting with extracellular ligands ensuring proper ligand availability at both short range and long range distances (Baeg et al., 2001; Filmus et al., 2008; Yan and Lin, 2009; Waghmare and Page-McCaw, 2022). Loss of *dlp* in a tissue impairs signaling at both short and long range (Desbordes and Sanson, 2003; Baeg et al., 2004; Kreuger et al., 2004; Franch-Marro et al., 2005; Yan et al., 2009; Zhang et al., 2013; Chaudhary et al., 2019).

Several transgenic flies overexpressing GFP-tagged *dlp* have been previously generated to elucidate Dlp’s function *in vivo* (Baeg et al., 2001; Han et al., 2004; Kreuger et al., 2004; Marois et al., 2006; Gallet et al., 2008). However, only a few of tagged constructs have been tested for functionality and only in a tissue-specific context, and at least one *GFP-dlp* transgene expressed in flies has been previously reported to be non-functional (Baeg et al., 2001; Han et al., 2004; Waghmare et al., 2020) . The use of non-functional tagged protein constructs can obscure our understanding of its function. For example, previous studies that have used different tagged-Dlp constructs have arrived at contradictory findings regarding its role in transcytosis of Wingless ligand from apical to basal surfaces in the epithelial cells (Gallet et al., 2008; Yan et al., 2009) . Such contradictions in the published literature underscore the importance of assessing whether or not tagged proteins expressed *in vivo* are functional.

In 2014, a large-scale protein trap screen using a hybrid *piggyBac* vector isolated a fly line that produced StrepII-Venus-StrepII (SVS)-tagged Dlp from the endogenous locus (Lowe et al., 2014) . The StrepII tag can be used for affinity purification of the tagged protein, and Venus is a fluorophore derived from GFP that allows protein visualization using fluorescence microscopy ( Nagai et al., 2002; Maertens et al. 2015) . The *piggyBac* insertion in the first intron contains an artificial exon that encodes SVS flanked by strong splice acceptor and donor sequences from the *Myosin heavy chain* ( *Mhc* ) gene. The *SVS-dlp* protein trap line is healthy and homozygous viable, and SVS-tagged Dlp can be detected in tissues isolated from *SVS-dlp* flies ( Fig. 1A ) (Lowe et al., 2014; Norman et al., 2016; Su et al., 2018; Waghmare et al., 2020) . Because *dlp* loss-of-function is embryonic lethal (Baeg et al., 2001; Han et al., 2004) , the homozygous protein trap line must produce a functional protein. While this is a reasonable assumption, it comes with a caveat. It is possible that the protein-trap produces wild-type *dlp* isoform through alternative splicing ( Fig. 1B ), contributing to the survival of the homozygous flies. This potential issue may be circumvented by using CRISPR-Cas9 to delete the endogenous locus followed by ФC31 integrase-mediated knock-in of tagged protein sequence. Indeed, a recent study has generated a fly line that expresses YFP-tagged *dlp* from endogenous locus using this strategy (Ell et al. 2024) . This line, however, is not homozygous viable, and therefore likely non-functional. These data further suggest that the position of the tag within a protein likely affects its function and underscore the importance of determining if the existing endogenously-tagged *SVS-dlp* line is functional. Standard genetic complementation assays cannot test this possibility. Therefore, we used Reverse Transcriptase- Polymerase Chain Reaction (RT-PCR) to test if the *SVS-dlp* flies produce the wild-type isoform. RT- PCR generates amplicons using cDNA as a template generated from the mRNA isoforms present in the sample using reverse transcriptase. Thus, the cDNA acts as a proxy for the mRNA isoforms present in the sample. We designed forward and reverse primers against sequences in exons 1 and 2 respectively that generate the expected 475bp amplicon when *UAS-dlp* plasmid is used as template ( Fig. 1C ). These primers are also able to amplify the 475bp amplicon from wild-type *dlp* isoform and the 1282bp amplicon from the *SVS-dlp* isoform present in the samples isolated from fly tissues ( Fig. 1D ). Our results show that the primers can detect the wild-type *dlp* isoform in *w ^1118^* flies but this isoform is not detected in SVS-Dlp flies. Further, the same primers detect the *SVS-dlp* isoform in SVS-Dlp flies. Because SVS-Dlp flies do not produce detectable wild-type *dlp* isoform to contribute to their survival, we conclude that the *SVS-dlp* isoform is functional.

## Methods

Total RNA was isolated from adult *w ^1118^* (control) and *SVS-dlp* ( *PBac{602.P.SVS-1}dlp ^[CPTI000445]^* ) protein-trap line using the RNeasy Mini Kit (Qiagen) per manufacturer’s protocol. 6-8 adult male and female flies were used. The RNA sample was treated with DNAse to remove any possible genomic DNA contamination during RNA isolation. SuperScript IV VILO kit (Invitrogen) was used to synthesize cDNA from RNA. PCR was performed using Q5 polymerase (NEB) using the manufacturer’s protocol. DNA gel electrophoresis was performed on PCR products, which were separated using ethidium bromide-containing 1% agarose gel. The *UAS-dlp* plasmid used in this study contains wild-type *dlp* sequence cloned into pUAST vector and was obtained from Xinhua Lin.

Immunohistochemistry was performed on age-matched seven-day old female flies using previously published protocol (Waghmare et al., 2020) . Briefly, ovaries were dissected in ice-cold PBS (Phosphate buffered saline), fixed in 4% paraformaldehyde for 20 minutes as room temperature, washed with 1X PBST (PBS plus 0.1% Triton X-100), and incubated for 1h in 5% NGS (normal goat serum in 1X PBS). After blocking, tissues were incubated overnight with mouse IgG1 anti-Dlp (13G8; 1:5) from Developmental Studies Hybridoma Bank (DSHB) and rabbit anti-GFP (Abcam ab6556; 1:200). Following 3 washes with 1xPBST, samples were were incubated with Cy3-conjugated goat anti-mouse IgG1 (115–165-205) and FITC-conjugated goat anti-rabbit (# 711-095-152) secondary antibodies for 1h in dark at room temperature (1:500, Jackson Immuno Research). DAPI was used to stain nuclei (1 μg/ml), and the samples were mounted in Vectashield (Vector Laboratories). Images were captured using Zeiss Apotome Imager M2 under identical settings, and figures were generated using Affinity designer and Affinity Photo.

## Reagents

**Table:** 

**Fly line**	**Genotype**	**Source**	**Stock**
Control	* w ^1118^*	Bloomington stock center	BDSC_3605
*SVS-dlp*	* w ^1118^ ; PBac{602.P.SVS-1}dlp ^CPTI000445^*	Kyoto stock center	115031
**Primers**	**Sequence**	**Source**	
Forward primer	ATGCTACATCAGCAGCAACA	Generated in this study	
Reverse primer	CTTTGAATTTTGTGGCCTTG	Generated in this study	
